# Early vascular toxicity induced by *Bothrops atrox* venom in the chorioallantoic membrane assay: Kinetic profile and translational insights

**DOI:** 10.1371/journal.pntd.0013969

**Published:** 2026-02-02

**Authors:** Hatem Kallel, Marwa Lakhrem, Zakaria Boujhoud, Sanah Essayagh, Said Hilali, Manel Mallouli, Marwa Bouhamed, Majed Kammoun, Dabor Resiere, Jean Marc Pujo, Stéphanie Houcke, Ibtissem Ben Amara

**Affiliations:** 1 Intensive Care Unit, French Guiana University Hospital, Cayenne, French Guiana; 2 Tropical Biome and immunopathology CNRS UMR-9017, Inserm U 1019, Université de Guyane, Cayenne, French Guiana; 3 Laboratory of Medicinal and Environmental Chemistry, Higher Institute of Biotechnology, University of Sfax, Sfax, Tunisia; 4 Laboratory of Health Sciences and Technologies, High Institute of Health Sciences, Hassan 1st University, Settat, Morocco; 5 Laboratory of Health and Food, Faculty of Science and Technology, Settat, Morocco; 6 Laboratory of Anatomopathology, CHU Habib Bourguiba, University of Sfax, Sfax, Tunisia; 7 Intensive Care Unit, Martinique University Hospital, Fort de France, Martinique; 8 Emergency Department, French Guiana University Hospital, Cayenne, French Guiana; Universidade Federal do Amazonas, BRAZIL

## Abstract

**Background:**

Snakebite envenoming is a neglected tropical disease with significant morbidity and mortality, particularly in areas with limited resources. *Bothrops atrox* is the most important snake involved in human envenomings in the Amazon. Its venom induces complex vascular damage that contributes to hemorrhage and systemic complications. This study employs the chicken chorioallantoic membrane (CAM) model to illustrate and quantify the acute vascular toxicity of *B. atrox* venom.

**Methodology/principal findings:**

Fertilized chicken eggs at embryonic day 9 were exposed to escalating doses of *B. atrox* venom (1, 50, and 100 µg/egg) for up to 300 seconds. Vascular alterations were assessed using macroscopic imaging, quantitative analysis with ImageJ and AngioTool, and histological examination. Venom exposure resulted in dose- and time-dependent vascular disruption, mainly vascular rupture and hemorrhage. At low doses, we observed minimal hemorrhage without any significant changes in vascular network architecture. At high doses, histopathology revealed endothelial disorganization, vessel dilation, leukocyte infiltration, and microthrombi formation, consistent with direct cytotoxic and inflammatory effects.

**Conclusions/significance:**

*B. atrox* venom rapidly compromises vascular integrity and triggers an inflammatory response in the CAM model, reflecting key pathophysiological features of envenomation. The severity of these effects was proportional to the duration of exposure and the venom dose used. These findings support the use of CAM assay as a translational tool for screening venom-induced vascular toxicity and underscore the imperative of early antivenom administration.

## Author summary

Snakebite is a major health problem, especially in tropical regions. *Bothrops atrox* is a common snake in South America. Its venom can cause significant tissue damage, including vascular injury. We used the chicken egg chorioallantoic membrane (CAM) model, a cost-effective tool, to illustrate the early vascular toxicity induced by *B. atrox* venom. We exposed the CAM to different amounts of *B. atrox* venom for short periods. Using imaging techniques, we observed that the venom rapidly damages the blood vessels, causing them to leak and become disorganized. The severity of this damage was proportional to the amount of venom and the time of exposure. We also found evidence of an inflammatory reaction in the affected vessels. These findings demonstrate that the CAM model is helpful in studying the early stages of venom-induced blood vessel damage. This model could be used in future research to compare venom toxicities, test the neutralisation potency of different antivenoms, and explore the specific mechanisms by which *B. atrox* venom acts.

## 1. Introduction

Snake bites are a significant public health issue globally, particularly in limited-resource areas, where they may lead to a substantial number of disability-adjusted life years if not adequately managed. In South America, *Bothrops atrox* is responsible for the majority of snakebites [1,2]. Its venom can affect blood clotting and exacerbate bleeding following vascular injury [3]. Additionally, it can trigger inflammatory response and redox imbalances, leading to systemic manifestations [4].

*B. atrox* venom contains a wide array of toxin families, including metalloproteinases (SVMPs), serine proteases (SVSPs), phospholipases A_2_ (PLA_2_s), hyaluronidases (Hyals), L-amino acid oxidases (L-AAOs), disintegrins, and many others. SVMPs are classified into three structural subclasses (PI, PII, and PIII) based on their domain composition [5]. SVMP-PIII, the main metalloproteinase contained in *B. atrox* venom, can target the microvasculature and degrade the components of the capillary basement membrane and disrupt cell-cell adhesion, leading to loss of vascular integrity and subsequent extravasation [6–8]. PLA_2_s contribute to cytotoxicity by disrupting membrane integrity and triggering an inflammatory response, which in turn increases vascular permeability and cell death [9]. The L-AAO from *B. atrox* venom can trigger cellular apoptosis through the generation of hydrogen peroxide (H_2_O_2_) during enzymatic activity [10]. As a result, SVMP-PIII, PLA_2_s, and L-AAO exhibit anti-angiogenic properties [11]. Disintegrins are small, non-enzymatic proteins that interfere with integrin-mediated cell adhesion, thereby affecting angiogenesis and endothelial integrity [12,13]. Their interference with platelet aggregation exacerbates systemic coagulopathy and amplifies systemic bleeding. Additionally, synergistic interactions among these major toxins further potentiate the venom’s overall pathogenic effects.

Preclinical animal models are routinely used to replicate the vascular toxicity induced by snake venom. However, ethical, financial, and technical challenges limit their use. In addition, contemporary research increasingly adheres to the ‘3Rs’ principle (replacement, reduction, and refinement) of animal experimentation. The chicken chorioallantoic membrane (CAM) is the outermost extra-embryonic membrane of fertilized chicken eggs. It is a highly vascularized model offering a robust, cost-effective alternative for evaluating vasoactive and angiogenic processes [14]. Extensive validation supports the CAM’s relevance as a preclinical and translational screening tool, with studies demonstrating its histological and toxicological parallels to animal models in studying vascular toxicity of venoms from *Bitis parviocula*, *Bitis arietans*, *Crotalus viridis*, *Agkistrodon contortrix*, *Crotalus atrox,* and *Crotalus adamanteus* [15–17]. However, a dedicated investigation into the specific vascular toxicity profile of *B. atrox* venom using this model remains unexplored, providing the basis of this research.

This work aimed to illustrate and quantify the early toxicity of *B. atrox* venom at varying doses and time points on the vascular network and the endothelial cells using the chicken CAM assay.

## 2. Materials and methods

### 2.1. Venom

*B. atrox* venom of adult specimens was purchased from Latoxan (Portes-lès-Valence, France).

### 2.2. The CAM-test principle

#### 2.2.1. Egg collection and incubation.

Freshly fertilized eggs of uniform weight were obtained from the agri-food company “Eurafric Poussins” in Had Soualem, Morocco (ICE: 001527398000095). Eggs were disinfected with 10% povidone-iodine and incubated for 72 h at 37 °C, with a relative humidity of 55–65%, and manual rotation every 3 h. On the fourth day, fertilized eggs were identified using transillumination and subsequently processed [14,15]. The eggshells were pierced at both poles using a sterile syringe and needle (20G). We removed 3 mL of albumen to free up space for experimental procedures. Punctures in the eggshell were then sealed with paraffin, and the eggs were returned to the incubator under the standard conditions as stated above.

#### 2.2.2. Experimental procedures.

**Fig 1:**
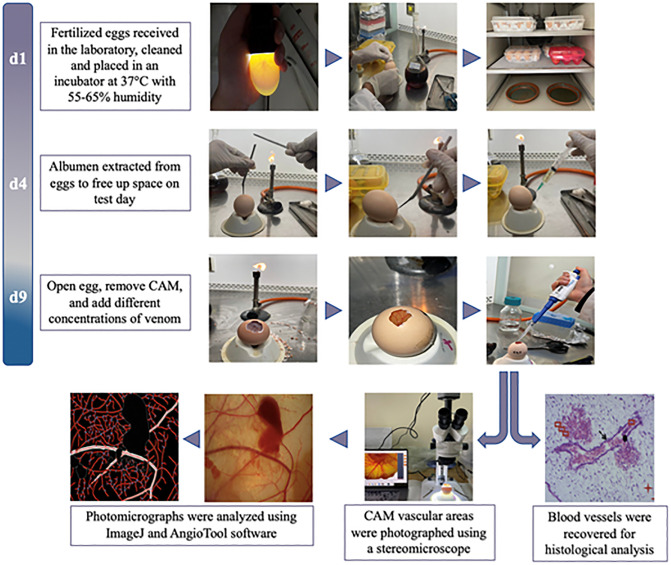
Experimental workflow for fertilized egg preparation and processing to assess the vascular effects of *Bothrops atrox* venom on the chorioallantoic membrane. The timeline reflects incubation days (d1–d9).

The eggs were removed from the incubator on the ninth embryonic day (9ED). Each egg was cleaned, and a 1.5 cm x 1.5 cm window was opened, revealing the CAM. Unfertilized eggs and non-viable embryos were discarded at this stage. A subset of eggs (n = 6) served as controls, and 50 μL of sterile Phosphate Buffered Saline (PBS; pH 7.2) was applied directly onto the CAM vessels of each one. Testing groups consisted of 6 eggs per venom dose and per time point. We used three amounts of venom (1, 50, and 100 μg/egg) dissolved in 50 µL PBS and applied to the surface of the vessels. Each egg from the control and testing groups was evaluated individually after application. Photographs were taken at 0, 30, 60, 90, 120, 180, and 300 seconds to monitor the vascular response. In total, 24 eggs were investigated per experiment. Fig 1 provides a detailed visual representation of the experimental workflow for fertilized egg preparation and processing.

#### 2.2.3. Quantitative analysis of the vascular network.

The photomicrographs obtained from the CAM vessels were analyzed using ImageJ (version 1.50i, National Institutes of Health, USA). To quantify the topography of the vascular network, areas of 500 mm^2^ were selected in the software. The images were then converted to grayscale, and inverted using the “Edit” plug-in. Background subtraction was performed by setting a threshold of 50 pixels in the “Process” plug-in.

Further analysis was conducted using the AngioTool software (version 0.6a, National Cancer Institute, USA) to evaluate vascular network parameters. Metrics included vessel area, total vessel length, number of junctions, and lacunarity (defined as the gaps between vessels). Results were expressed as percentages relative to the control group.

#### 2.2.4. Histological examination.

Blood vessels from the CAM were extracted, fixed in 10% formaldehyde, dehydrated, and embedded in paraffin. Slides were prepared with 5 μm sections and stained with hematoxylin and eosin. Histological images were captured using an optical microscope (Olympus BX43, Olympus Corporation, Japan) equipped with a digital camera (Olympus DP27). Observations were performed at 20× and 40 × magnifications.

### 2.3. Statistical analyses

Results are expressed as mean±SD. Bivariate statistical comparisons were assessed using the Mann-Whitney U Test. A *p-value* ≤0.05 was considered significant. Statistical analyses were carried out using IBM SPSS Statistics for Windows, version 24 (IBM Corp., Armonk, N.Y., USA).

## 3. Results

### 3.1. Hemorrhagic activity

**Fig 2:**
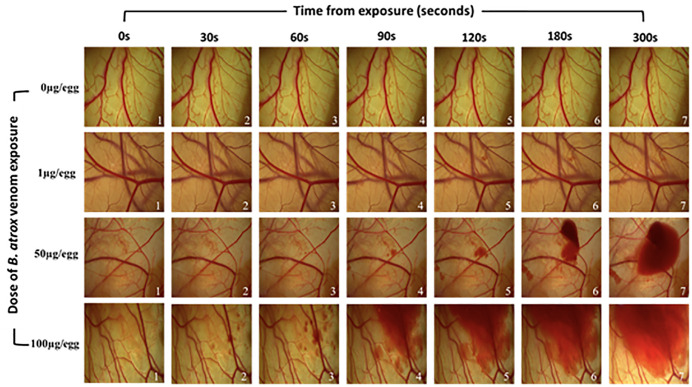
Evaluation of the effect of increasing *Bothrops atrox* venom doses on the chicken egg chorioallantoic membrane vascular network over time.

**Fig 3:**
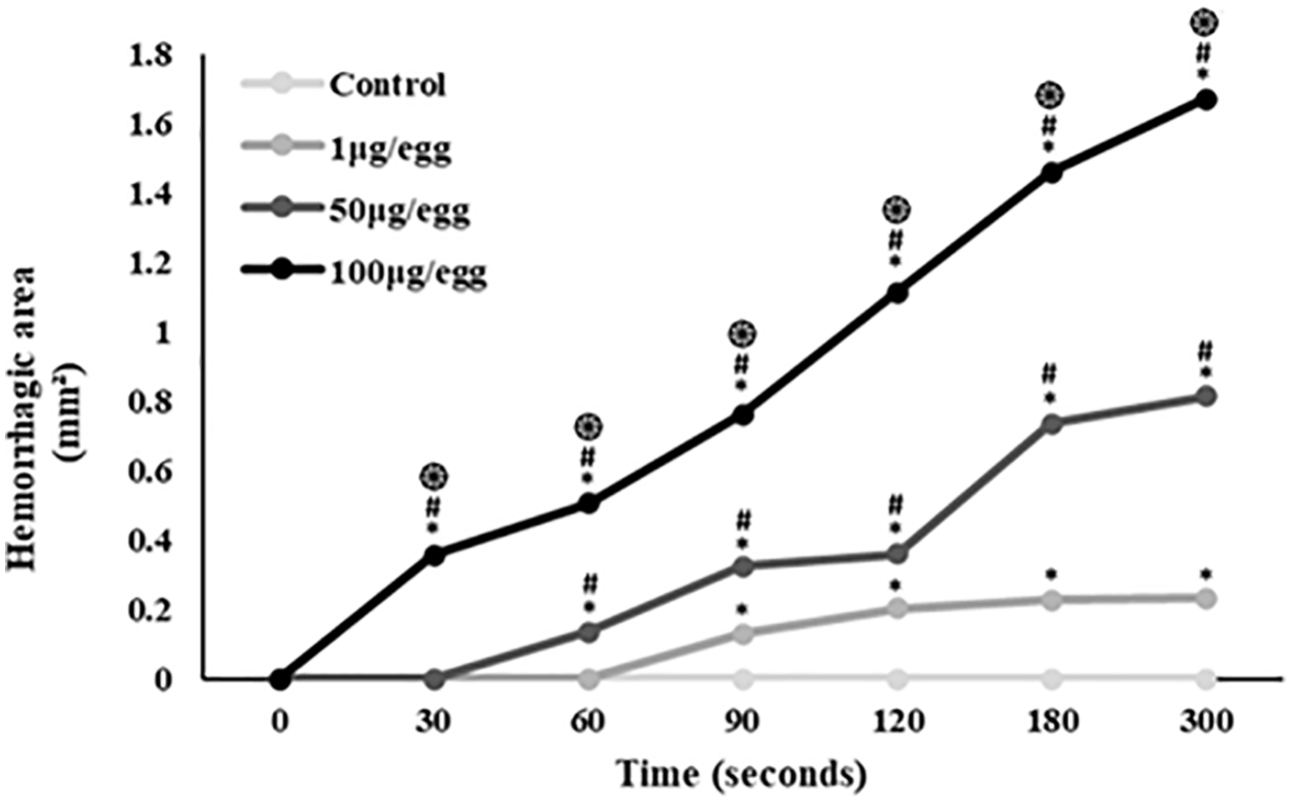
The mean hemorrhagic area induced by various *B. atrox* venom doses at different time points. * indicate significance compared to controls; # indicate significance compared to the 1 µg/egg group; ֍ indicate significance compared to the 50 µg/egg group.

CAM exposed to PBS showed a standard vascular network without hemorrhagic activity. At 1 µg venom/egg, the CAM exhibited slight blood extravasation starting at 90s. Application of 50 µg venom/egg resulted in hyperemia and enlargement of smaller capillaries, with hemorrhage noted from 60s. At 100 µg venom/egg, a small hemorrhage site appeared after 30s, with increased bleeding and a larger affected area after 180s of exposure. Fig 2 illustrates the effect of *B. atrox* venom on the CAM vascular network over time (0 to 300s). Fig 3 illustrates the progression of the hemorrhagic area over 300s, refining the macroscopic observed results. Increased amounts of *B. atrox* venom induced a significant increase in hemorrhage area.

### 3.2. Quantitative analysis of vascular network

**Fig 4:**
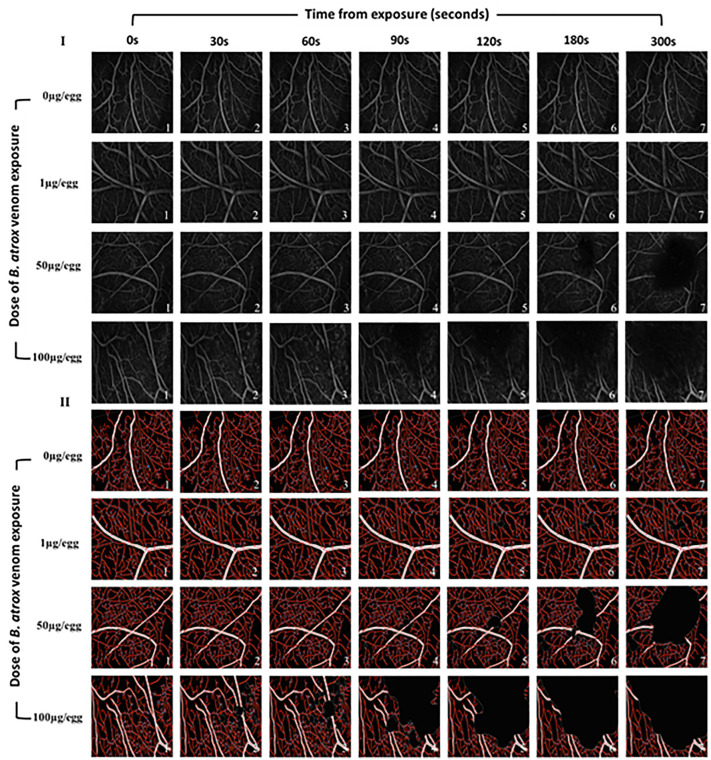
Analysis of vascular network architecture in chicken chorioallantoic membrane vascular network exposed to increasing doses of *B. atrox* venom over time. (I): A typical image processed on ImageJ v 1.50i software; (II): The vascular network architecture processed on AngioTool v 0.6a software. Ring fields were photographed at ×10 magnification.

**Fig 5:**
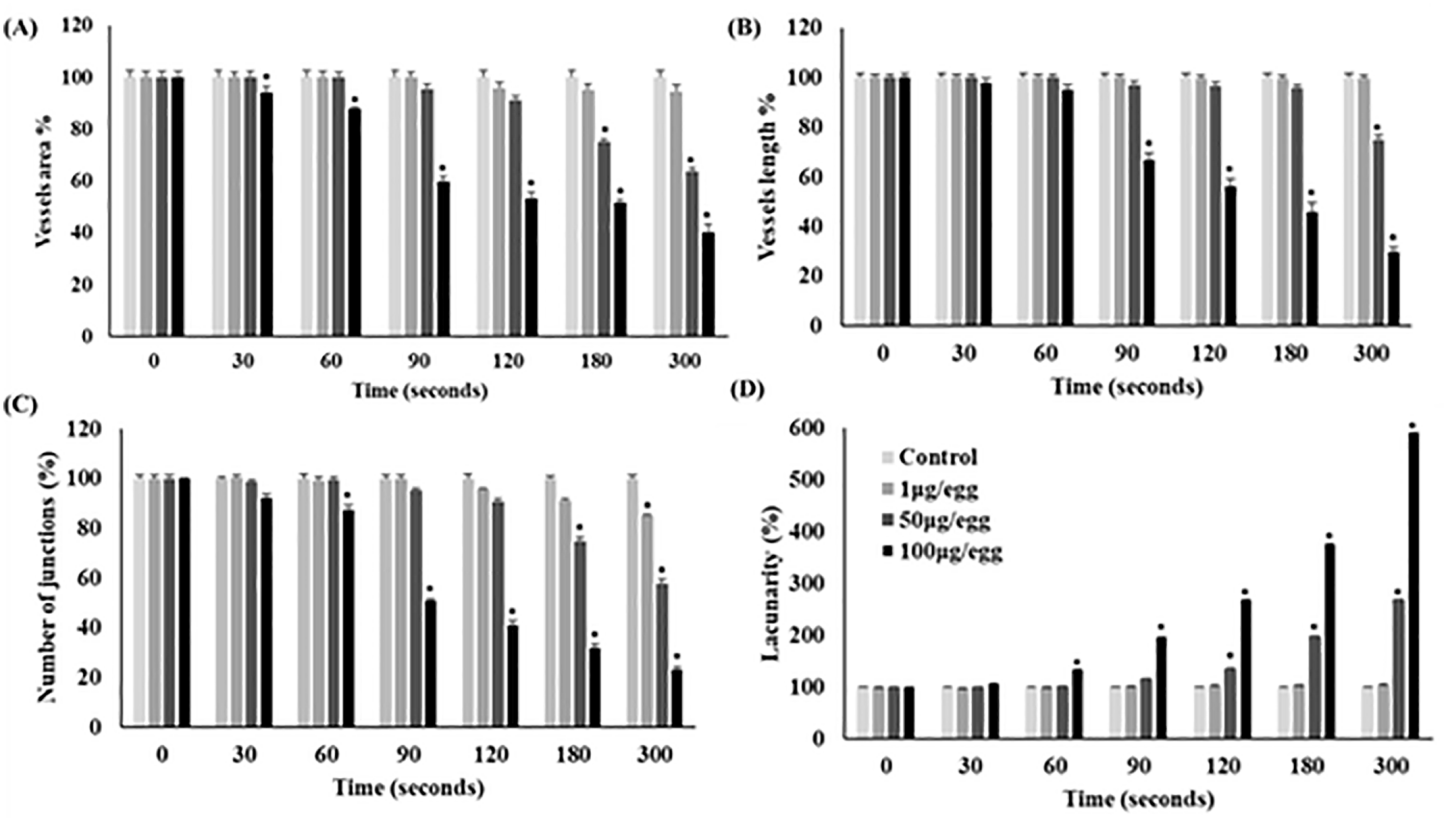
Evaluation of the effect of various *B. atrox* venom doses on the structural parameters of the CAM vascular network of the 9-day-old egg. Data are presented as percentages and standard deviation, with reference values (time 0s) set at 100%. (A): Vessels area, (B): Vessels length, (C): Junction number, and (D): Lacunarity. *p < 0.05 in comparison to the time point = 0 seconds for the same venom dose used.

The topography of the CAM vessel network after treatment with varying amounts of *B. atrox* venom is depicted in Fig 4. Quantitative analysis revealed that in areas with minimal hemorrhage (1 µg of venom from 0 to 300 seconds and 50 µg of venom from 0 to 120 seconds), no significant changes in vascular network parameters were observed (Figs 4 and 5). However, vessel area significantly decreased with 100µg venom/egg starting at 30s (-6%), and with 50µg venom/egg from 180s (-25%) (Fig 5A). Vessel length decreased significantly with 100µg venom/egg from 90s (-33%), and with 50µg venom/egg at 300s (-25%) (Fig 5B). The number of junctions significantly decreased with 100µg venom/egg from 60s (-12.4%), with 50µg venom/egg from 180s (-25%), and with 1µg venom/egg from 300s (-15%) (Fig 5C). Lacunarity significantly increased with 100µg venom/egg from 60s (+33.9%) and with 50µg venom/egg from 120s (+34.5%) (Fig 5D). These changes were proportional to the hemorrhagic area observed in Figs 2 and 3.

### 3.3. Histological sections of blood vessels

**Fig 6:**
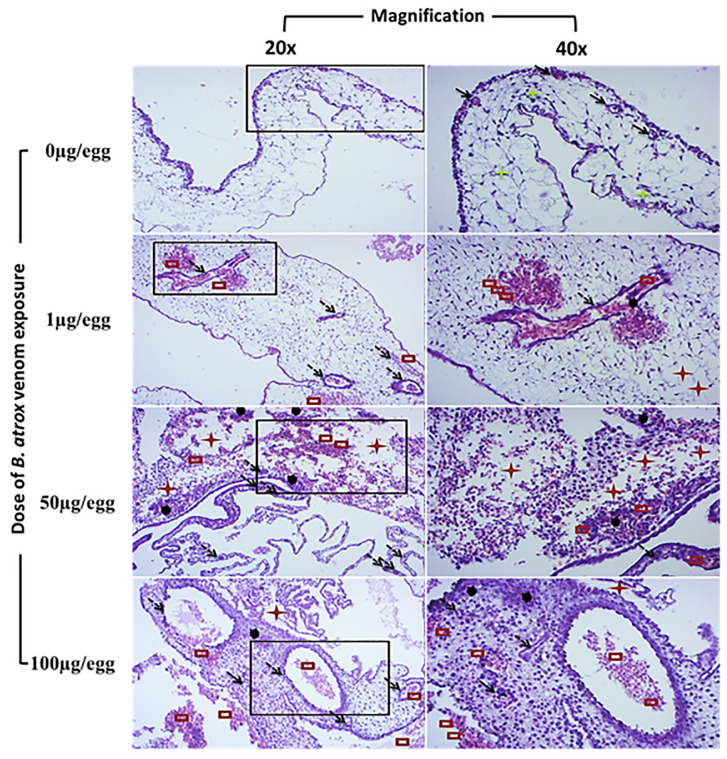
Histological sections stained with hematoxylin-eosin from 9ED chorioallantoic membranes treated with increasing doses of *Bothrops atrox* venom for 300s. **Symbols indicate:** 
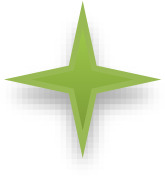
 Normal tissue, 
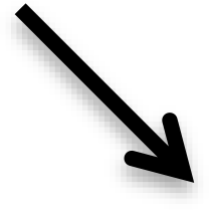
 Blood vessel, 
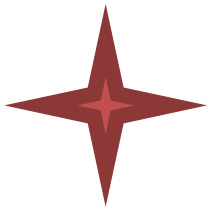
 Edema, 
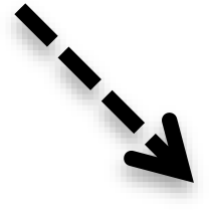
 Engorged blood vessel, 
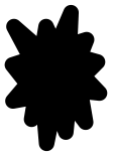
 Necrosis, 
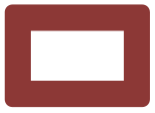
 Hemorrhage and leukocyte infiltration.

Histological examination of the CAM from control eggs showed standard vascular structure, well-defined blood vessels, and intact vessel walls. Eggs treated with 1µg venom showed subtle vascular alterations, including slight vessel dilation, partial endothelial cell disorganization, minimal leukocyte infiltration, and occasional microthrombi. Treatment with 50 µg venom/egg resulted in more pronounced vascular changes, dilated and tortuous vessels, severely disorganized endothelial cells, moderate leukocyte infiltration, and larger microthrombi. Treatment with 100 µg venom/egg led to severe vascular damage, characterized by highly dilated and tortuous vessels, weakened vascular walls, dense leukocyte infiltration, hemorrhage, and cell necrosis. Histological sections from the CAM’s blood vessels exposed to increasing *B. atrox* venom doses for 300s are presented in Fig 6.

## 4. Discussion

Using the CAM model, our study illustrates the early toxic effects of *B. atrox* venom on the vascular network. These effects associate direct toxic damage with leukocyte accumulation, indicating a robust immune response. In addition, we demonstrate the dose- and time-dependent toxic effects of the venom on the vessel and endothelial cells.

This study examined the CAM vasculature at 9ED, a developmental stage characterized by dynamic vascular proliferation resulting in an extensive and accessible arteriovenous system [14,18]. At this phase, mesenchymal tissue exhibits rigorous angiogenic activity, with a pronounced density of small-caliber vessels localized near the chorionic epithelium. Simultaneously, the mesodermal layer exhibits the formation of larger vessels, indicative of advancing vascular maturation. The well-formed blood network of the 9ED CAM is readily visible due to its transparent matrix, making it ideal for studying blood vessels [14]. Given these advantages, the CAM model has been employed to evaluate the vasoactive properties of six snake venoms [15–17]. Notably, Knight et al. showed that the venom of *Bitis arietans*, *Crotalus viridis*, and *Agkistrodon contortrix* result in hemotoxic effect and vascular irritation [15]. Petrilla et al. showed that venoms from *Bitis arietans* and *B. parviocula* are responsible for vasoactive effects and hemotoxicity [16]. Finally, Bekešová et al. documented the embryotoxic and vasoactive properties of *Crotalus atrox* and *C. adamanteus* venoms using the CAM model [17]. Adding *B. atrox* to that list expands the phylogenetic scope of venom studies. Additionally, elucidating the toxic vascular effects of *B. atrox* venom on the CAM vessels yields preliminary results that could pave the way for further research. Our study used the CAM model as a translational step to investigate the vascular toxicity, kinetics, and dose-response relationship of *B. atrox* venom in a controlled setting, offering foundational insights for future mechanistic and therapeutic investigations.

Our study investigated the architecture and distribution of CAM vessels following incubation with varying quantities of *B. atrox* venom (1, 50, and 100 μg/egg) across multiple time points. CAMs treated with 50 and 100 μg venom exhibited marked vascular damage. Substantial reductions in vessel area, vessel length, junction counts, and lacunarity were detected by AngioTool only in regions with extensive hemorrhagic foci. This finding is likely attributable to the fact that hemorrhagic zones are opaque under optical microscopy, preventing AngioTool from assessing the underlying vascular network. By contrast, in areas with minimal hemorrhage (1 µg of venom from 0 to 300 seconds and 50 µg of venom from 0 to 120 seconds), no significant changes in vascular-network parameters were observed. Collectively, these results indicate that the acute vascular toxicity of the venom is driven primarily by endothelial damage, rather than by disruption of the overall vascular architecture. Histological examination revealed dilated and tortuous vessels, weakened vascular walls, leukocyte infiltration, hemorrhage, and cellular necrosis. These pathological changes are consistent with the known actions of *B. atrox* venom components. Indeed, *B. atrox* venom contains 101 distinctive proteins mainly represented by SVMPs (40.2%), PLA_2_s (19.5%), SVSPs (11%), L-AAO (7.3%), C-type lectins (7.3%), and disintegrins (2.4%) [3]. These toxins exert, independently or synergistically, direct toxic effects by degrading the components of the capillary basement membrane and disrupting cell-cell adhesion. Beyond its direct toxic effects, *B. atrox* venom triggers a complex inflammatory response and redox imbalance. This response involves early plasma exudation (bradykinin, histamine), polymorphonuclear cell migration (C3a, C5a, IL-8), endothelial activation (selectins via histamine, TNF-α), and direct tissue disruption by venom enzymes [19]. Specifically, BaTX-II, an Asp49 PLA2 isolated from *B. atrox* venom, can induce hydrogen peroxide production by neutrophils, stimulate inflammatory mediators (e.g., IL-1β, IL-8, LTB4), and trigger neutrophil extracellular trap formation [20]. In a murine model, *B. atrox* venom causes local hemorrhage, myonecrosis, mast cell degranulation, and macrophage phagocytosis, leading to superoxide production by migrated neutrophils and organ injury [21]. Although oxidative stress in the CAM vessels is not thoroughly investigated, Easterling *et al.* [22] demonstrated that L-AAO-generated reactive oxygen species can mediate cell death in the chicken CAM. In addition, histological findings indicate an active inflammatory response. Although the chicken immune system is not fully developed at 9ED [14], our results suggest that inflammation, potentially mediated by PLA_2_, may contribute to the pathogenesis of the vascular effects. Indeed, venom administered to the CAM can reach the systemic circulation, affecting the embryo and triggering immune and inflammatory responses [23]. Our study was conducted on 9ED, prior to the full maturation of the chicken immune system [14]. However, granulopoiesis initiates on the seventh embryonic day [24] and significant recruitment of granulocytic leukocytes to inflammation sites can be detected from this stage [25]. Moreover, cytokines and chemokines like IL-1β, IL-8, IL-12, and IL-18 are produced from ED3 [24]. Overall, the vascular damage observed on the CAM treated with *B. atrox* venom could be explained by direct toxicity, redox disturbances, and an active inflammatory response [4].

Our study demonstrates that vascular damage induced by *B. atrox* venom follows a time-dependent pattern. This aligns with experimental findings where the toxicity of *B. atrox* venom follows an increasing time-dependent trend of manifestations, including hemorrhagic activity [26]. This also mirrors clinical observations where envenoming symptoms can worsen within the first 24 hours post-bite [2,27]. These convergent results underscore the need for rapid antivenom (AV) administration to neutralize circulating venom and prevent the escalation of toxic effects in envenomed victims [2,27–29]. However, given our limited observational window to 300s, longer follow-up is necessary to fully elucidate venom kinetics and pinpoint when vascular damage stabilizes, potentially signaling the cessation of venom activity and reduced benefit of delayed antivenom in clinical practice. Moreover, our study found that vascular damage progresses in proportion to the venom dose. A similar result was observed in mice, confirming that severity correlates with the amount of venom injected [26]. Also, severity grading systems likely reflect the circulating venom load in a clinical setting [30]. This aligns with the principle that “*it is the dose that makes the poison*.” Collectively, these findings support the relationship between venom quantity, clinical manifestations, and the necessity for higher AV doses in severe cases.

We acknowledge several potential limitations in this study. First, while more advanced *in vitro* models exist for assessing vascular dynamics and hematotoxicity, we used the CAM assay due to its cost-effectiveness, accessibility, and well-documented utility in evaluating acute venom-induced vascular effects [15–17]. Second, although the exact composition of the *B. atrox* venom batch used was not fully characterized, its overall enzymatic profile is well-established, with only potential variations in the relative abundance of individual components [3,31]. Third, our observational window was limited to 300s, which constrains our ability to fully capture the long-term kinetics and progression of venom-induced vascular damage. Nonetheless, this timeframe was sufficient to document acute vascular effects and reveal time- and dose-dependent patterns. Fourth, this study relies on semi-automated quantification using AngioTool software to investigate the early vascular response to snake venom. It is important to note, that our investigation focused on early vascular toxicity, with an observation window limited to 5 minutes. Such a brief interval is unlikely to capture meaningful changes in angiogenesis. Studies incorporating longer observation periods will be required to characterize the sustained vascular effects of the venom and its potential impact on vascular network architecture. To our knowledge, this is the first application of AngioTool software in the context of snakebite venom research. Fifth, we are aware that the small sample size (n = 6 per group) limits the power of our analysis. We therefore used the Mann-Whitney U test, a non-parametric test well-suited for small sample comparisons. While this approach is statistically sound, we interpret our findings cautiously, presenting results with a significance threshold of p ≤ 0.05, rather than reporting exact *p-values*, to reflect the exploratory nature of these analyses. Despite these limitations, our preliminary findings provide a valuable foundation for developmental vascular toxicology and support future investigations to elucidate mechanistic pathways and enhance translational relevance to mammalian systems.

## 5. Conclusion

This study used the CAM model to document the kinetics of early toxic vascular effects induced by *B. atrox* venom. We observed a complex response, including variations in vascular diameter, endothelial damage, and high leukocyte infiltration, indicative of an intense inflammatory reaction following *B. atrox* venom exposure. Finally, the observed time- and dose-dependent toxicity gives insight into the importance of administering antivenom early and at the appropriate dose for *B. atrox* envenoming victims.
